# Towards the virtual artery: a multiscale model for vascular physiology at the physics–chemistry–biology interface

**DOI:** 10.1098/rsta.2016.0146

**Published:** 2016-11-13

**Authors:** Alfons G. Hoekstra, Saad Alowayyed, Eric Lorenz, Natalia Melnikova, Lampros Mountrakis, Britt van Rooij, Andrew Svitenkov, Gábor Závodszky, Pavel Zun

**Affiliations:** 1 Computational Science Laboratory, Institute for Informatics, Faculty of Science, University of Amsterdam, Sciencepark 904, 1098 XH Amsterdam, The Netherlands; 2 High Performance Computing Department, ITMO University, Saint Petersburg, Russia; 3 King Abdulaziz City for Science and Technology (KACST), Riyadh, Saudi Arabia; 4 Electric Ant Lab BV, Panamalaan 4 K, 1019AZ Amsterdam, The Netherlands

**Keywords:** virtual artery, multiscale model, arterial physiology

## Abstract

This discussion paper introduces the concept of the *Virtual Artery* as a multiscale model for arterial physiology and pathologies at the physics–chemistry–biology (PCB) interface. The cellular level is identified as the mesoscopic level, and we argue that by coupling cell-based models with other relevant models on the macro- and microscale, a versatile model of arterial health and disease can be composed. We review the necessary ingredients, both models of arteries at many different scales, as well as generic methods to compose multiscale models. Next, we discuss how this can be combined into the virtual artery. Finally, we argue that the concept of models at the PCB interface could or perhaps should become a powerful paradigm, not only as in our case for studying physiology, but also for many other systems that have such PCB interfaces.

This article is part of the themed issue ‘Multiscale modelling at the physics–chemistry–biology interface’.

## 1. Introduction

Professor J. P. Boon defined the physics–chemistry–biology (PCB) interface as ‘PCB Interface = mesoscopic domain connecting micro and macro scales' (J. P. Boon 2016, private communication, Solvay Workshop on Bridging Gaps at the PCB Interface). We would like to add to this definition that the PCB interface is also about connecting some B (biology) at one spatio-temporal scale with some P (physics) and/or C (chemistry) at the same or other spatio-temporal scales. As we will argue in this discussion paper, the notion of coupling B at the mesoscale with P and/or C at other scales is very important when aiming to model (human) physiology. The paradigm we propose is to pick the single cell at the mesoscale as the starting point for multiscale models of physiology [1], and then in a ‘middle-out-fashion’ [2] couple relevant processes on the larger tissue/organ scales or smaller intra-cell/molecular scales [3].

The *Virtual Artery* is a multiscale model at the PCB interface. The mesoscopic level represents relevant single-cell processes both in the arterial wall (endothelial cells, smooth muscle cells (SMCs)) and in the flowing blood (red blood cells (RBC), platelets, white blood cells (WBC)). The virtual artery relies on our generic multiscale modelling and simulation framework (MMSF) [4] applied to biomedicine [3,5], also including a multiscale computing environment [6–8]. In this discussion paper, we first provide a compact review of modelling and simulation of arterial physiology and pathologies, only referring to review papers covering major topics. The original research and papers are discussed in those reviews. We will also shortly review the MMSF and finally discuss how all these components can be synthesized into the virtual artery.

## 2. Modelling arterial physiology and pathologies

‘You are as young as your arteries' (J. Gunn 2010, private communication, Sheffield, UK), a very true quote indeed, indicating that, as the most prevalent cause of death is due to cardiovascular disease [9], for many of us the health condition of our vascular tree is an important determinant of our expectancy for a healthy and active life. The arterial tree is a very complex branching structure equipped with an equally complex pump, the heart, circulating an equally complex fluid, the blood, transporting oxygen, nutrients and wastes to and from all tissues in the body [10,11]. Blood flow in arteries is pulsatile and under normal conditions laminar (except in the ascending aorta) with complex secondary flows in branches and curves [12,13]. Arteries are living organs and are capable in many ways of adapting to changing physiological conditions in order to meet changing demands in, for example, oxygen [10,11,14].

Blood flow exerts a wall shear stress (WSS) on the monolayer of endothelial cells lining the inside of the arterial wall. These endothelial cells can sense the WSS and use that signal for a wide repertoire of biochemical responses [13]. Also the SMCs in the tunica media are capable of sensing and reacting to pressure changes of blood through interstitial flow [13]. The pulsatile nature of blood flow, in combination with the tortuous and branching structure of the arterial tree can create local haemodynamic environments with low oscillatory WSS changing direction during every cardiac cycle. These sites, such as the carotid bifurcation, tend to develop atherosclerotic lesions [13,14]. Note that atherosclerosis at the carotid bifurcation is the main cause of stroke [14]. More generally, as Tarbell *et al.* [13] write: ‘Atherosclerosis is a disease of the arterial wall and thrombotic events caused by it are by far the most common cause of death in the world’. An atherosclerotic lesion can develop into a restriction in the blood vessel, a stenosis, which has many effects on the local flow dynamics [15]. This may lead to destabilization and possible rupture of plaques [16]. High shear stresses at a stenosis can also trigger platelet activation and thrombosis, which can then lead to an embolism that can totally block upstream arteries, such as coronary arteries (leading to a heart attack) or arteries in the brain (leading to stroke) [14]. The interplay between the mechanical forces due to flowing blood and the biological response on the molecular level (pathways, gene expression, etc.) receives a lot of attention. For instance, the process of mechanotransduction, its influence on gene expression and subsequent implications for arterial responses, albeit still far from understood, are now being unravelled in much detail [13]. Another major vascular disorder are aneurysms, which mainly appear in the abdominal and thoracic aorta and the vessels in the brain along the circle of Willis [17,18]. Again, an intricate interplay between pulsating blood pressure and shear stresses exerted on the wall and biological processes in the arterial wall lead to degenerative changes and finally to permanent dilations that we call aneurysms. When left untreated they have a probability of rupturing, leading to acute life-threating bleeding in the brain or abdomen [14,17–19].

Because of its essential role in maintaining a healthy vessel and in the development of diseases, haemodynamics has, therefore, become a major field of study. Because *in vivo* measurement of blood flow remains a non-trivial procedure, even in state-of-the-art medical imaging devices, computational haemodynamics has been and remains a very important tool to study and understand haemodynamics [13,18,20–22]. Many arterial systems have been studied in detail, e.g. flow in coronary arteries [23,24], as well as blood flow in stenosed arteries [15], aneurysms [17–19] or other abnormal situations [25]. Similarly, the effects of treatment of arterial disease on blood flows are studied in much detail, e.g. after stenting abdominal aortic aneurysms [26], or after stenting of stenosed coronary arteries [27]. With this knowledge, it should be possible to optimize treatments and indeed some very good examples have appeared in the recent literature [28].

Next, we consider blood itself, which of course is not a continuous fluid. It is a suspension of RBC, platelets and WBC in plasma, which itself is a protein rich fluid [10,11]. Besides being the transporter for oxygen and nutrients, it also carries all major components for haemostasis and thrombosis (the platelets and proteins in the plasma), and for the immune response (the WBCs, and many factors in the plasma). We will not further discuss the immune system, admittedly one of the most complex systems invented by nature. We merely note that in principle transport of WBCs and inflammatory and immune responses can also be taken into account in the virtual artery as proposed below.

There have been many studies on the biomechanics of single RBCs and how they behave in flows. This has led to a powerful set of models capable of simulating the behaviour of single RBCs as well as suspensions of many RBCs in vessels [29]. One important function of blood falling into the range of these studies is the haemostatic response, where a delicate balance exists between halting of bleeding by forming haemostatic thrombi, while at the same time avoiding vascular occlusion [30]. Many details are still unknown [31], with open questions ranging from, for example, the physics of blood rheology and cell margination to the effect of local flow conditions on the expression and interaction of membrane and plasma proteins in haemostasis, and there is a clear role for *in silico* modelling to support this type of research [32–34]. There are many papers in the literature proposing to model haemostasis and thrombosis at many different scales, using quite a broad range of methodologies [35].

## 3. The multiscale modelling and simulation framework

In earlier projects, we have developed the so-called MMSF for designing, implementing and executing multiscale applications [3,4,7,36–41]. This framework has been successfully tested on applications from several fields of science and technology (e.g. fusion, computational biology, biomedicine, nanomaterial science, rheology of complex fluids and hydrology). The MMSF offers many benefits: a clear methodology, software and algorithm reuse, the possibility to couple new and legacy codes, heterogeneous distributed computing and access to unprecedented computing resources [4].

The MMSF views a multiscale model as a set of coupled single-scale models, where the couplings (the scale-bridging methods) can be as simple as interpolations or amount to complete models by themselves. A multiscale model is quantitatively expressed as a set of single-scale models on a scale separation map (SSM), with directional edges between single-scale models to represent scale bridging. This leads to a classification of the multiscale model, to computing paradigms and to execution profiles [4,36]. The main distinction between multiscale models is loosely coupled (acyclic) versus tightly coupled (cyclic) [4,36]. In loosely coupled multiscale models, feedback between single-scale models is absent. Tightly coupled models, on the other hand, contain feedback loops. A microscale model could feed for instance information to a macroscale model, which uses this microscopic information to take one time step on that macroscale, and then a further microscale simulation is performed, feeding the next time step on the macroscale, etc. We have formally captured such tightly coupled multiscale models in terms of a generic submodel execution loop, and couplings between a small number of generic operators [36], leading to only four coupling templates. The multiscale modelling language (MML) translates all these concepts into a graphical (gMML) and machine-readable (xMML) specification of the multiscale model. These representations contain in principle sufficient information for the execution of the multiscale model in any type of computing environment. Moreover, the xMML description also represents a qualitative description of the multiscale model, and can be used to share a multiscale model with collaborators, or as a scaffold to create more detailed models when needed. For more details and a theoretical underpinning, we refer the reader to Borgdorff *et al.* [36]. A final important ingredient is the implementation of the single-scale models representing all relevant processes, as well as the scale-bridging methods to glue them together. Many models of that kind are available in the context of arterial health and disease, as discussed in the previous section.

**Figure 1.**
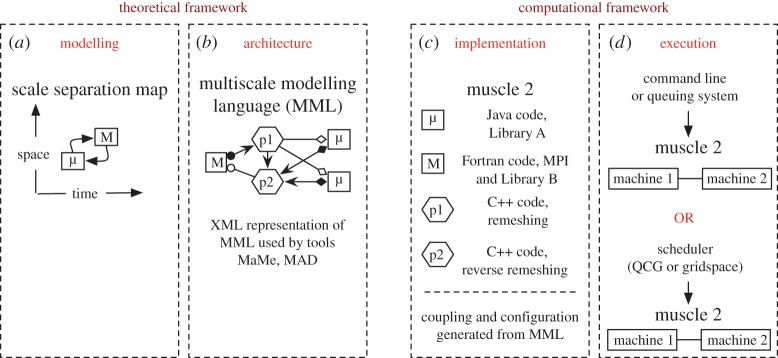
The MMSF pipeline. (*a*) *Modelling*: identification of the scales of the subprocesses and their relation. (*b*) *Architecture*: identification and definition of information workflow/dependencies between submodels using MML. (*c*) *Implementation* of coupling and communication between submodels and data processors/mappers (M) using MML and coupling libraries such as MUSCLE2. (*d*) Actual *execution* of the coupled models on possibly distributed and heterogeneous computing resources using orchestration/coordination tools.

The MMSF, therefore, is a methodological and practical way to model, characterize and simulate multiscale phenomena. MMSF currently comprises a four-stage pipeline, going from developing a multiscale model to executing a multiscale simulation (figure 1). We describe MMSF in detail in [4,36]. We are currently developing the MMSF in two directions, first with high-performance computing (HPC) capabilities and with sensitivity analysis and uncertainty quantification. There is a strong need to find generic ways to execute multiscale simulations on current petascale and emerging exascale computing systems. We call this high-performance multiscale computing. The ComPat project (www.compat-project.eu) will significantly contribute to this need by developing new technologies based on its vision of multiscale computing patterns.

## 4. Towards the virtual artery

The premise of the virtual artery is to start with models of individual cells and their interactions. For instance, the mechanical behaviour of single RBC and platelets was modelled using a boundary element method and coupled with flow of plasma using the immersed boundary method [42,43]. This was then used to study the transport of platelets in aneurysms [44] as well as the shear-induced diffusion of RBC [45]. So far, these models are purely P (physics) models. Currently, we are adding biology and chemistry to capture processes in relation to thrombosis. Platelets are then equipped with models of receptors, platelets can be activated and then bind to each other or to the arterial wall. Another example is our model of the arterial wall, more specifically the tunica media in coronary arteries, in relation to modelling in-stent restenosis (ISR) [46,47], which is an unwanted growth of tissue in a coronary artery after treatment of a stenosis with balloon angioplasty and stenting. The SMCs are the main culprit in this response, because their migration and proliferation lead to neointima formation [47,48]. The individual SMCs are modelled as agents which mechanically interact with each other via repulsive and attractive forces, while at the same time each cell goes through a cell cycle containing switches taking the cell's mechanical and biochemical situation into account, eventually leading to either apoptosis or proliferation [46]. This model of the tunica media is embedded in an overall multiscale model for ISR [47] which has been applied to the study of many aspects of this pathology [47–51]. Another important cell type in this response is the endothelial cell. Details of re-endothelialization of the damaged vessel wall are key to understanding the dynamics of the restenosis response, as was demonstrated by taking endothelial cells explicitly into account in the multiscale model, and subsequently formulating and testing a number of hypotheses for the dynamics of ISR [49,51].

The next step is to couple the cell-level processes to other relevant processes, typically operating on other spatio-temporal scales. For instance, the agent-based model for the tunica media in the ISR example discussed above could be coupled to a continuum mechanics model to capture the behaviour of the adventia. Or, as is already done in the ISR model, a continuous blood flow model is used to compute local WSSs on the endothelial cells, which use this signal in a model of NO production that regulates the proliferation of the SMCs [49]. Such models, in turn, can then be coupled again to other processes.

**Figure 2.**
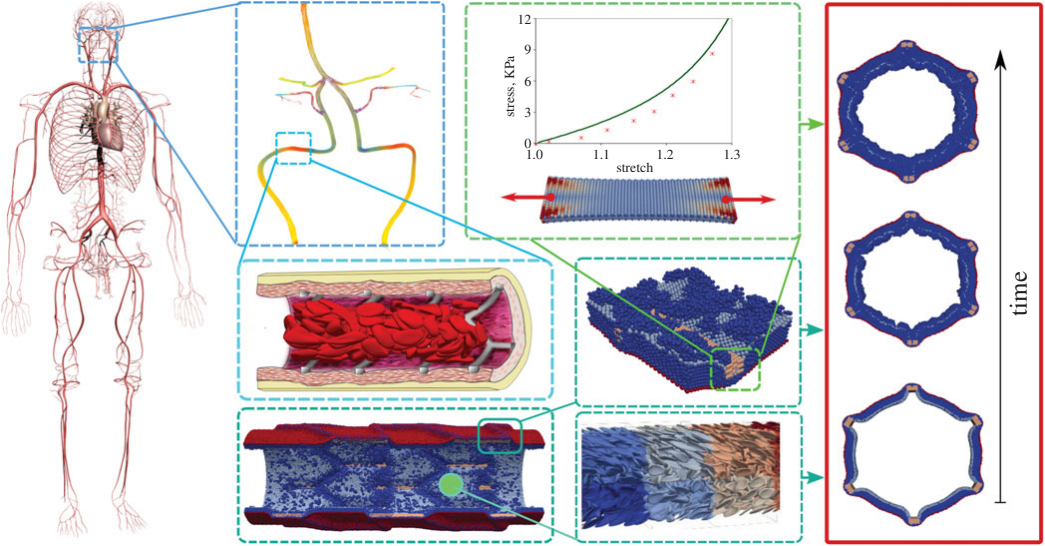
Towards the *Virtual Artery*. A combination of models on several scales that make up the virtual artery, ranging from whole-body one-dimensional models, via three-dimensional fully resolved haemodynamics, to cell-based models of blood and the arterial wall and intra-cellular processes.

We believe that many of the bits-and-pieces (the single-scale models that capture relevant processes on a range of spatio-temporal scales, from the organism level down to the intra-cellular level) are available or will become available in the near future, allowing us to create the virtual artery, a multiscale model for healthy and pathological arteries. Figure 2 shows a diagram of the components such as we believe they will appear in the model. The virtual artery will be driven by one-dimensional whole-body models [52], providing boundary conditions to image-based three-dimensional models of blood flow. These are in turn coupled to cell-based models, such as in [29,43]. More detailed models can be added when needed. Over the years, we have developed many components which, once integrated, together form the virtual artery: whole-body models [53], three-dimensional haemodynamics, based on the lattice Boltzmann method [54,55], cellular Potts models for the arterial wall [56], agent-based models of the tunica media [46] (see also [57]), cell-based models for blood [42–45] and cell-based models for platelet aggregation and thrombosis [58]. In the literature, many more models are described as discussed in the previous sections. We have also demonstrated that complex arterial pathologies can be modelled and simulated using the MMSF. We believe this is a clear demonstration of the potential of the approach, a strong motivation for further effort in the development of a complete virtual artery framework, and an illustration of future potential use in detailed studies of arterial pathologies.

Following the virtual physiological human (VPH) vision, the virtual artery is not ‘the supermodel’, it is a way to share observations, to derive predictive hypotheses and to integrate them into a constantly improving understanding of human physiology and pathology and to apply this in real clinical settings [59].

An important ingredient is the PCB interface. While at the mechanical level we can forgo the cellular nature of the arteries (blood included), at the PCB interface we cannot ignore the cellular composition of the arterial elements and the cross-regulatory role that these models have with each other. In the example of thrombosis, the endothelium plays a crucial role in the release of NO, which is also dependent on the shear stress the walls are experiencing. We already have several arterial-related models; coupling them will not only potentially yield more accurate results, but also a better understanding of the underlying phenomena and the improvement of the single-scale models.

The virtual artery will be a set of MMSF definitions (processes, scale bridging) specified in an xMML file and graphically available as a SSM or as a gMML rendering, not necessarily with actual implementations of all single-scale submodels and scale-bridging methods. An example of such SSM for a very detailed description of ISR is available in [47] and more examples are discussed in [3]. The MMSF virtual artery skeleton should be publically available, such that in a community-wide effort, researchers use and contribute to the virtual artery. Note that the CellML initiative has achieved such status [60] and the CellML philosophy and approach will be a guiding principle for the virtual artery*.* Next, single-scale models can actually be plugged into (parts) of the virtual artery, thus building specific models for specific phenomena to be studied. The single-scale models could also be in the public domain, as part of the virtual artery, but alternatively could also be proprietary codes. Other researchers could use their own submodel implementations and validate the coupling and the choice of the relevant processes independently (also depending on what problem they want to study). The addition of increasing numbers of implementations of single-scale models, but also execution environments, and sufficient data for validation and parameter tuning, renders the virtual artery into a community-driven model allowing the study of many aspects of healthy and pathological arteries.

## 5. Discussion

This paper discusses our vision for creating the virtual artery, a collection of MMSF specifications, single-scale models and scale-bridging methods that when taken together allow multiscale simulation of arterial health and pathologies at the PCB interface. In the spirit of the VPH, it will become a model structure, expressed in MML, and it will have a whole series of single-scale models and scale-bridging methods to glue them together. Our approach will be, in a community-wide effort, to build up the virtual artery from specific systems that we study, and at the same time to create the virtual artery SSM. In time, the virtual artery will provide an open infrastructure, allowing for an organized community effort. We expect that the virtual artery can have strong added value and impact, not only in cardiovascular research, but also in supporting treatment, and for health and well-being in general.

As one of us discussed in an earlier position paper [61], multiscale modelling and simulation remains to be a challenging activity with many open questions, both conceptually and in practice. Core issues relate to scale-bridging methods for the virtual artery, and how they, in turn, will impact validation, error propagation, verification and consistency of the simulations. Some relevant scale-bridging methods are well understood, e.g. coupling three-dimensional to one-dimensional/zero-dimensional haemodynamics models [62]. Others are not that well developed or do not exist yet, e.g. coupling cell-based blood flow models to three-dimensional continuous fluid models, or coupling cell-based models of the arterial wall to continuous solid mechanical models. More fundamental questions could also be asked. Can we find underlying theories that allow generic PCB scale-bridging methods between cell-based models and other, usually continuous models? Would the paradigm of the PCB interface allow us to take the underlying physics, chemistry and biology into account in some formal way, maybe formulating minimal sets of conservation laws that could exist at the PCB interface? In other words, can we find generic scale-bridging methods at the PCB interface?

Validation of multiscale models, and error propagation, verification and consistency of multiscale simulations are largely unexplored areas [61]. Moreover, sensitivity analysis and uncertainty quantification, which in themselves are quite mature fields [63,64], have hardly been applied to multiscale modelling. Yet, as with many models at the PCB interface, the virtual artery will be composed of a combination of physical, chemical and biological models. One may state that usually physical and chemical models have a known range of validity, and that uncertainty in those models is known. For biological models, that is already a much harder statement. By coupling single-scale models together additional uncertainty will be introduced, both directly through coupling parameters and indirectly through the enormous increase in complexity in which any small submodel uncertainty or spurious numerical artefact might build up to significant deviations on the scale of the coupled system. Physiological processes will usually have quite a large number of parameters. Most of these parameters are not known accurately, due to, for example, biological variability, or because they were measured in animals but not in humans, etc. Therefore, the sensitivity to each of these parameters should be well understood, and given their sensitivity and accuracy, a thorough uncertainty quantification should then result in reliable error bars on the simulations results. Certainly, when the virtual artery would be used in predictive scenarios, e.g. in *in silico* clinical trials or decision support systems, such uncertainty quantification should be a standard procedure. At the moment, no widely accepted theory on uncertainty propagation in multiscale models at the PCB interface exists. For the virtual artery to be of any predictive power, its development needs to go hand in hand with such efforts.

Finally, while some of the models can be computationally rather cheap, cell-based models usually are quite computationally intensive. That means, in practice, virtual artery simulations will most likely require high-end computing capabilities and in some cases even a heterogeneous HPC environment, depending on requirements of single-scale models. As an example for typical computational demand of a submodel, we know that cell-based blood flow simulations need computing power of the order of petaflop(s) and beyond (e.g. [43]). We have demonstrated before how typical multiscale models of human physiology can rely on advanced multiscale computing paradigms to achieve optimal computational efficiency [6,8], and we are currently extending the MMSF with capabilities for high-performance multiscale computing (in the ComPat project, see www.compat-project.eu). Moreover, virtual artery simulations will typically require not one single run, but extended parameter range studies (e.g. for population studies in an *in silico* clinical trial scenario or for uncertainty quantification), adding to their expected high computational costs. Our vision is to embed the virtual artery into a computational structure (embodied by the MMSF) that should in principle facilitate multiscale computing at high-end machines, thus taking away the burden of orchestrating such non-trivial computing jobs from the scientists who develop virtual artery models and apply them to study vascular (patho)physiology. Great progress has been achieved towards this goal; still, the level of proved maturity of such computational environments needed for clinical application will demand further great development effort.

The virtual artery, like many if not most multiscale models of physiology, fits the concept of models at the PCB interface. We believe that this concept, as introduced and discussed during the Solvay workshop ‘Bridging the gaps at the PCB interface’ and laid down in many of the papers in this theme issue of *Philosophical Transactions A*, can become a very powerful paradigm. And not only a paradigm where the gaps between P, B and C are bridged, but also a paradigm where a broad range of multidisciplinary scientists meet, interact and make progress. It is at the interface between the sciences where much exiting research is possible, and models at the PCB interface hold this promise. Seen in that light, the virtual artery is just an example of the tremendous opportunities offered by the paradigm of models at the PCB interface.

## References

[1] Walker DC, Southgate J 2009 The virtual cell---a candidate co-ordinator for ‘middle-out’ modelling of biological systems. Br. Bioinform. 10, 450–461. (10.1093/bib/bbp010) 19293250

[2] Noble D 2012 A theory of biological relativity: no privileged level of causation. Interface Focus 2, 55–64. (10.1098/rsfs.2011.0067) 23386960 PMC3262309

[3] Sloot PMA, Hoekstra AG 2010 Multi-scale modelling in computational biomedicine. Br. Bioinform. 11, 142–152. (10.1093/bib/bbp038) 20028713

[4] Chopard B, Borgdorff J, Hoekstra AG 2014 A framework for multi-scale modelling. Phil. Trans. R. Soc. A 372, 20130378 (10.1098/rsta.2013.0378) 24982249 PMC4084523

[5] Hoekstra AG, Chopard B, Lawford P 2014 Multiscale modelling and simulation. In Computational biomedicine, modelling the human body (eds Coveney P, Diaz-Zuccarini V, Hunter P, Viceconti M). Oxford, UK: Oxford University Press.

[6] Groen D et al. 2013 Flexible composition and execution of high performance, high fidelity multiscale biomedical simulations. Interface Focus 3, 20120087 (10.1098/rsfs.2012.0087) 24427530 PMC3638484

[7] Borgdorff J, Mamonski M, Bosak B, Groen D, Belgacem MB, Kurowski K, Hoekstra AG 2013 Multiscale computing with the multiscale modeling library and runtime environment. Proc. Comput. Sci. 18, 1097–1105. (10.1016/j.procs.2013.05.275)

[8] Borgdorff J et al. 2014 Performance of distributed multiscale simulations. Phil. Trans. R. Soc. A 372, 20130407 (10.1098/rsta.2013.0407) 24982258 PMC4084531

[9] World Health Organization. See http://www.who.int/mediacentre/factsheets/fs310/en/index2.html.

[10] Levick JR 2010 An introduction to cardiovascular physiology, 5th edn Boca Ratton, FL: CRC Press.

[11] Boron WF, Boulpaep EL 2012 Medical physiology, 2nd edn Philadelphia, PA: Saunders Elsevier.

[12] Ku DN 1997 Blood flow in arteries. Annu. Rev. Fluid Mech. 29, 399–434. (10.1146/annurev.fluid.29.1.399)

[13] Tarbell JM, Shi Z-D, Dunn J, Jo H 2014 Fluid mechanics, arterial disease, and gene expression. Annu. Rev. Fluid Mech. 46, 591–614. (10.1146/annurev-fluid-010313-141309) 25360054 PMC4211638

[14] Wootton DM, Ku DN 1999 Fluid mechanics of vascular systems, diseases, and thrombosis. Annu. Rev. Biomed. Eng. 1, 299–329. (10.1146/annurev.bioeng.1.1.299) 11701491

[15] Berger SA, Jou L 2000 Flows in stenotic vessels. Annu. Rev. Fluid Mech. 32, 347–382. (10.1146/annurev.fluid.32.1.347)

[16] Stoll G, Bendszus M 2006 Inflammation and atherosclerosis novel insights into plaque formation and destabilization. Stroke 37, 1923–1932. (10.1161/01.STR.0000226901.34927.10) 16741184

[17] Lasheras JC 2007 The biomechanics of arterial aneurysms. Annu. Rev. Fluid Mech. 39, 293–319. (10.1146/annurev.fluid.39.050905.110128)

[18] Humphrey JD, Taylor CA 2008 Intracranial and abdominal aortic aneurysms: similarities, differences, and need for a new class of computational models. Annu. Rev. Biomed. Eng. 10, 221–246. (10.1146/annurev.bioeng.10.061807.160439) 18647115 PMC2742216

[19] Sforza DM, Putman CM, Cebral JR 2009 Hemodynamics of cerebral aneurysms. Annu. Rev. Fluid Mech. 41, 91–107. (10.1146/annurev.fluid.40.111406.102126) 19784385 PMC2750901

[20] Taylor CA, Draney MT 2004 Experimental and computational methods in cardiovascular fluid mechanics. Annu. Rev. Fluid Mech. 36, 197–231. (10.1146/annurev.fluid.36.050802.121944)

[21] Heil M, Hazel AL 2011 Fluid-structure interaction in internal physiological flows. Annu. Rev. Fluid Mech. 43, 141–162. (10.1146/annurev-fluid-122109-160703)

[22] Taylor C, Steinman D 2010 Image-based modeling of blood flow and vessel wall dynamics: applications, methods and future directions. Ann. Biomed. Eng. 38, 1188–1203. (10.1007/s10439-010-9901-0) 20087775

[23] Kim H, Vignon-Clementel I, Coogan J, Figueroa C, Jansen K, Taylor C 2010 Patient-specific modeling of blood flow and pressure in human coronary arteries. Ann. Biomed. Eng. 38, 3195–3209. (10.1007/s10439-010-0083-6) 20559732

[24] Melchionna S, Bernaschi M, Succi S, Kaxiras E, Rybicki FJ, Mitsouras D, Coskun AU, Feldman CL 2010 Hydrokinetic approach to large-scale cardiovascular blood flow. Comput. Phys. Commun. 181, 462–472. (10.1016/j.cpc.2009.10.017)

[25] Loth F, Fischer PF, Bassiouny HS 2008 Blood flow in end-to-side anastomoses. Annu. Rev. Fluid Mech. 40, 367–393. (10.1146/annurev.fluid.40.111406.102119)

[26] Kleinstreuer C, Li Z, Farber MA 2007 Fluid-structure interaction analyses of stented abdominal aortic aneurysms. Annu. Rev. Biomed. Eng. 9, 169–204. (10.1146/annurev.bioeng.9.060906.151853) 17362195

[27] Duraiswamy N, Schoephoerster RT, Moreno MR, Moore JE 2007 Stented artery flow patterns and their effects on the artery wall. Annu. Rev. Fluid Mech. 39, 357–382. (10.1146/annurev.fluid.39.050905.110300)

[28] Marsden AL 2014 Optimization in cardiovascular modeling. Annu. Rev. Fluid Mech. 46, 519–546. (10.1146/annurev-fluid-010313-141341)

[29] Freund JB 2014 Numerical simulation of flowing blood cells. Annu. Rev. Fluid Mech. 46, 67–95. (10.1146/annurev-fluid-010313-141349)

[30] Marder VJ, Aird WC, Bennet JS, Schulman S, White GC II 2012 Hemostasis and thrombosis, basic principles and clinical practice, 6th edn Baltimore, MD: Lippincott Williams and Wilkins.

[31] Esmon CT, Esmon NL 2011 The link between vascular features and thrombosis. Annu. Rev. Physiol. 73, 503–514. (10.1146/annurev-physiol-012110-142300) 20887194

[32] Welsh JD, Stalker TJ, Voronov R, Muthard RW, Tomaiuolo M, Diamond SL, Brass LF 2014 A systems approach to hemostasis: 1. The interdependence of thrombus architecture and agonist movements in the gaps between platelets. Blood 124, 1808–1815. (10.1182/blood-2014-01-550335) 24951424 PMC4162110

[33] Stalker TJ, Welsh JD, Tomaiuolo M, Wu J, Colace TV, Diamond SL, Brass LF 2014 A systems approach to hemostasis: 3. Thrombus consolidation regulates intrathrombus solute transport and local thrombin activity. Blood 124, 1824–1831. (10.1182/blood-2014-01-550319) 24951426 PMC4162112

[34] Welsh JD, Muthard RW, Stalker TJ, Taliaferro JP, Diamond SL, Brass LF 2016 A systems approach to hemostasis: 4. How hemostatic thrombi limit the loss of plasma-borne molecules from the microvasculature. Blood 127, 1598–1606. (10.1182/blood-2015-09-672188) 26738537 PMC4807424

[35] Fogelson AL, Neeves KB 2015 Fluid mechanics of blood clot formation. Annu. Rev. Fluid Mech. 47, 377–403. (10.1146/annurev-fluid-010814-014513) 26236058 PMC4519838

[36] Borgdorff J, Falcone J-L, Lorenz E, Bona-Casas C, Chopard B, Hoekstra AG 2013 Foundations of distributed multiscale computing: formalization, specification, and analysis. J. Parallel Distrib. Comput. 73, 465–483. (10.1016/j.jpdc.2012.12.011)

[37] Borgdorff J et al. 2012 A distributed multiscale computation of a tightly coupled model using the multiscale modeling language. Proc. Comput. Sci. 9, 596–605. (10.1016/j.procs.2012.04.064)

[38] Hoekstra A, Caiazzo A, Lorenz E, Falcone J-L, Chopard B 2010 Complex automata: multi-scale modeling with coupled cellular automata. In Simulating complex systems by cellular automata (eds Hoekstra AG, Kroc J, Sloot PMA), pp. 29–57. Berlin, Germany: Springer.

[39] Falcone J-L, Chopard B, Hoekstra A 2010 MML: towards a multiscale modeling language. Proc. Comput. Sci. 1, 819–826. (10.1016/j.procs.2010.04.089)

[40] Caiazzo A, Falcone J-L, Chopard B, Hoekstra AG 2009 Asymptotic analysis of complex automata models for reaction-diffusion systems. Appl. Numer. Math. 59, 2023–2034. (10.1016/j.apnum.2009.04.001)

[41] Hoekstra AG, Lorenz E, Falcone J-L, Chopard B 2007 Towards a complex automata framework for multi-scale modeling. Int. J. Multiscale Comput. Eng. 5, 491–502. (10.1615/IntJMultCompEng.v5.i6.60)

[42] Mountrakis L, Lorenz E, Hoekstra AG 2014 Validation of an efficient two-dimensional model for dense suspensions of red blood cells. Int. J. Mod. Phys. C 25, 1441005 (10.1142/S0129183114410058)

[43] Mountrakis L, Lorenz E, Malaspinas O, Alowayyed S, Chopard B, Hoekstra AG 2015 Parallel performance of an IB-LBM suspension simulation framework. J. Comput. Sci. 9, 45–50. (10.1016/j.jocs.2015.04.006)

[44] Mountrakis L, Lorenz E, Hoekstra AG 2013 Where do the platelets go? A simulation study of fully resolved blood flow through aneurysmal vessels. Interface Focus 3, 20120089 (10.1098/rsfs.2012.0089) 24427532 PMC3638486

[45] Mountrakis L, Lorenz E, Hoekstra AG 2016 Scaling of shear-induced diffusion and clustering in a blood-like suspension. Europhys. Lett. 114, 14002 (10.1209/0295-5075/114/14002)

[46] Caiazzo A et al. 2011 A complex automata approach for in-stent restenosis: two-dimensional multiscale modelling and simulations. J. Comput. Sci. 2, 9–17. (10.1016/j.jocs.2010.09.002)

[47] Evans DJW et al. 2008 The application of multiscale modelling to the process of development and prevention of stenosis in a stented coronary artery. Phil. Trans. R. Soc. A 366, 3343–3360. (10.1098/rsta.2008.0081) 18603527

[48] Tahir H, Hoekstra AG, Lorenz E, Lawford PV, Hose DR, Gunn J, Evans DJW 2011 Multi-scale simulations of the dynamics of in-stent restenosis: impact of stent deployment and design. Interface Focus 1, 365–373. (10.1098/rsfs.2010.0024) 22670206 PMC3262436

[49] Tahir H, Bona-Casas C, Hoekstra AG 2013 Modelling the effect of a functional endothelium on the development of in-stent restenosis. PLoS ONE 8, e66138 (10.1371/journal.pone.0066138) 23785479 PMC3681932

[50] Amatruda CM et al. 2014 From histology and imaging data to models for in-stent restenosis. Int. J. Artif. Organs 37, 786–800. (10.5301/ijao.5000336) 25044386

[51] Tahir H, Bona-Casas C, Narracott AJ, Iqbal J, Gunn J, Lawford P, Hoekstra AG 2014 Endothelial repair process and its relevance to longitudinal neointimal tissue patterns: comparing histology with *in silico* modelling. J. R. Soc. Interface 11, 20140022 (10.1098/rsif.2014.0022) 24621816 PMC3973369

[52] van de Vosse FN, Stergiopulos N 2011 Pulse wave propagation in the arterial tree. Annu. Rev. Fluid Mech. 43, 467–499. (10.1146/annurev-fluid-122109-160730)

[53] Svitenkov A, Rekin O, Hoekstra AG. Submitted. Sensitivity of hemodynamic simulations to the level of detail of the arterial tree model.

[54] Axner L, Hoekstra A, Jeays A, Lawford P, Hose R, Sloot P 2009 Simulations of time harmonic blood flow in the mesenteric artery: comparing finite element and lattice Boltzmann methods. Biomed. Eng. 8, 23 (10.1186/1475-925x-8-23) PMC2764713 19799782

[55] Artoli AMM, Hoekstra AG, Sloot PMA 2006 Mesoscopic simulations of systolic flow in the human abdominal aorta. J. Biomech. 39, 873–884. (10.1016/j.jbiomech.2005.01.033) 16488226

[56] Tahir H, Niculescu I, Bona-Casas C, Merks RMH, Hoekstra AG 2015 An *in silico* study on the role of smooth muscle cell migration in neointimal formation after coronary stenting. J. R. Soc. Interface 12, 20150358 (10.1098/rsif.2015.0358) 26063828 PMC4528603

[57] Melnikova N, Hoekstra AG. Submitted. A cell based mechanical model of coronary artery tunica media. 10.1098/rsif.2017.0028 PMC5550961 28679664

[58] Belyaev AV, Mountrakis L, Lorenz E, Panteleev MA, Hoekstra AG. Submitted. Three-dimensional simulations of blood platelets aggregation mediated by membrane adhesion potentials.

[59] STEP_Consortium. 2007 Seeding the EuroPhysiome: a roadmap to the virtual physiological human.

[60] Beard DA et al. 2009 CellML metadata standards, associated tools and repositories. Phil. Trans. R. Soc. A 367, 1845–1867. (10.1098/rsta.2008.0310) 19380315 PMC3268215

[61] Hoekstra A, Chopard B, Coveney P 2014 Multiscale modelling and simulation: a position paper. Phil. Trans. R. Soc. A 372, 20130377 (10.1098/rsta.2013.0377) 24982256

[62] Shi Y, Lawford P, Hose R 2011 Review of zero-D and 1-D models of blood flow in the cardiovascular system. Biomed. Eng. Online 10, 33 (10.1186/1475-925X-10-33) 21521508 PMC3103466

[63] Smith RC 2014 Uncertainty quantification: theory, implemenation, and applications. Philadelphia, PA: SIAM.

[64] Saltelli A et al. 2008 Global sensitivity analysis. The primer. New York, NY: John Wiley & Sons.

